# Haemorrhoidal disease in pregnancy: results from a self-assessment questionnaire administered by means of a social network

**DOI:** 10.1186/s12876-024-03228-5

**Published:** 2024-05-02

**Authors:** Angela D’Alfonso, Francesca De Carolis, Alessandro Serva, Sayali Valiyeva, Maurizio Guido, Renato Pietroletti

**Affiliations:** 1 Unit of Obstetrics and Gynaecology, Department of Life, Health and Environmental Sciences, University of L’Aquila, L’Aquila, Italy; 2 Surgical Coloproctology, University of L’Aquila, Hospital Val Vibrata, Sant’Omero, TE Italy; 3 Department of Clinical Sciences and Biotechnology, University of L’Aquila, Surgical Coloproctolgy Hospital Val Vibrata Sant’Omero, 64027 Sant’Omero, TE Italy

**Keywords:** Haemorrhoids, Pregnancy, Social media, Maternal health, Anal symptoms

## Abstract

**Background:**

The anal symptoms occurring during pregnancy and post-partum, mainly related to Haemorrhoidal Disease (HD), have been reported with in a wide range of incidence in the literature. Although in many cases the course of the disease is mild and self-limiting, sometimes it is severe enough to affect quality of life.

**Methods:**

Our study has been conducted through a questionnaire administered via social media with the aim of obtaining epidemiologic data on the incidence of the symptoms of HD in an unselected population of pregnant women. In addition, we looked for the presence of those factors notoriously predisposing or associated to HD (constipation, straining on the toilet, low dietary fibres and fluid intake).

**Results:**

Out of 133 patients 51% reported symptoms of HD during pregnancy, mainly in the second and third trimester. Constipation, straining on the toilet, low dietary fibres and fluid intake were not significantly related to incidence of HD. Only a previous history of HD was correlated to onset of symptoms of HD in pregnancy and reached a statistical significance (odds ratio = 5.2, *p* < 0.001).

**Conclusion:**

Although with the limitations posed by the nature of our retrospective study via a self-assessment interview, our results suggest that the occurrence of HD in pregnancy seems not sustained by the classical risk factors observed in the general population. At the moment, specific therapeutic measures are lacking and treatment relies on empiric suggestions concerning diet, fluid intake, bowel care, local ointment. Further studies are needed in order to identify a targeted etiologic treatment.

**Supplementary Information:**

The online version contains supplementary material available at 10.1186/s12876-024-03228-5.

## Background

Quality of life during pregnancy and immediately after delivery can be marred by anal symptoms, not rarely severe, related to hemorrhoidal disease (HD). The prevalence of HD in pregnancy is considered high with data from literature, mainly obtained by means of interview, ranging from 24 to 35% [1–4], up to an estimated peak about 85% [5] particularly in the 2nd and 3rd trimester. However, data on the true prevalence are limited and variably reported [1, 6]. Symptoms commonly associated with HD are bright red blood at defecation, anal pain, pruritus ani, mucus discharge and prolapse. Hemorrhoids can be divided into external and internal. These last are usually classified in four degrees, according to Goligher’s grading of prolapse. External haemorrhoids may develop a painful thrombosis, a frequent cause of consultation during pregnancy and after childbirth. HD and in less extent anal fissures [6] are certainly among the leading causes of rectal bleeding in pregnant women and a proctological examination can make a differential diagnosis [3, 7]. Different etiologic factors are deemed responsible for HD in pregnancy, such as increased pelvic pressure, obstruction to pelvic venous outflow, relaxing effect of progesterone on the venous wall, increased circulating blood volume and constipation [2, 8], with age and parity hypothesized as predisposing factors [3, 4]. A history of HD and constipation has been associated to HD in pregnancy but no association was found with gestational hormonal changes [9]. In a recent study by Bužinskiene et al., additional risk factors were identified, such as prolonged pregnancy (more than 40 weeks), prolonged labour or straining duration of more than 20 min, instrumental delivery and newborn weight of more than 3,800 g [10]. Interestingly in spite of this, guidelines for HD prevention and treatment during pregnancy are the same as those recommended in the general population and thus mainly focused on bowel function suggesting dietary changes, fluid and bran intake [11], however with controversial results [12]. Other treatments recommended consist of warm sitz baths or local ointment [13, 14], whereas flavonoids use is not supported, lacking evidence of their safety profile in pregnancy [4, 13–16].

Childbirth often represents the beginning of a spontaneous outburst of HD [17], but a high rate of thrombosed haemorrhoids is recorded in third trimester and after labour with a further worsening of symptoms (20% after labour, 7.8% in 3rd trimester) [18].

The self-assessment questionnaire has been the most frequently used instrument to assess symptoms of HD in pregnancy or after delivery [6]. However, this method, although very easy to perform, shows limitations in terms of accuracy [6, 19, 20], especially when adopted in hospital setting, selecting those patients seeking for medical advice.

Social media represent a large source of health information for pregnant women and an increasing use of these medias for self-monitoring of pregnancy and infant care has been reported, especially in low-income countries [21, 22].

In our work we adopted a questionnaire concerning symptoms of HD in pregnancy, launched in a widespread social media with the aim of investigate the occurrence of HD in pregnancy, in an unselected population of women answering spontaneously to the questionnaire. We aimed to investigate the role-played during pregnancy by all those well-known risk factors of HD for the general population. In addition, we investigated the relationship between delivery modality and HD. To our knowledge, this approach has never been adopted in studies concerning HD in pregnancy.

## Methods

For our purpose the questionnaire was published on the web by means of Google Modules, in different Facebook’s groups concerning pregnancy, maternal health, delivery experience. In our patients’ sample, only primiparous women were considered in order to exclude those women with a history of perianal disorders of obstetric origin. We aimed to observe a direct effect of pregnancy on the development of HD and perianal symptoms [23]. The questionnaire is reported in detail in the Appendix 1. We did not collect any personal information in the questionnaire in order to guarantee anonymity.

The comparison between women who complained of HD in pregnancy and those who did not was performed by Chi-square or Fischer tests. All variables were analysed in a multiple logistic regression model with Confidence Interval (CI) settled at 95%. Statistical software adopted was The R Foundation for Statistical Computing, R version 3.3.2 (2016), Sincere Pumpkin Patch.

## Results

One hundred and thirty-seven women answered to the questionnaire; 4 were excluded due to incongruent or incomplete answers. Out of 133 primiparous women, 65 did not report any anal symptom during pregnancy whereas 68 complained of anal symptoms referred as HD that makes 51% of the cases. Table 1, shows incidence according to the period of pregnancy or delivery; the 2nd and 3rd trimester accounted for more than 70% of the reported cases, with 57.3% in the 3rd trimester.

**Table 1:** Occurrence of HD in pregnancy in 68 symptomatic women and reasons for not seeking medical advice

Occurrence of HD	N.O (%)
*First trimester*	*8 (11.7)*
*Second trimester*	*11 (16.1)*
*Third trimester*	*39 (57.3)*
**Reasons for not** **Seeking medical advice**	
*Embarrassment*	*18 (54.5)*
*Early Ealing*	*10 (30.3)*
*Lack of time*	*6 (18.2)*

**Fig. 1:**
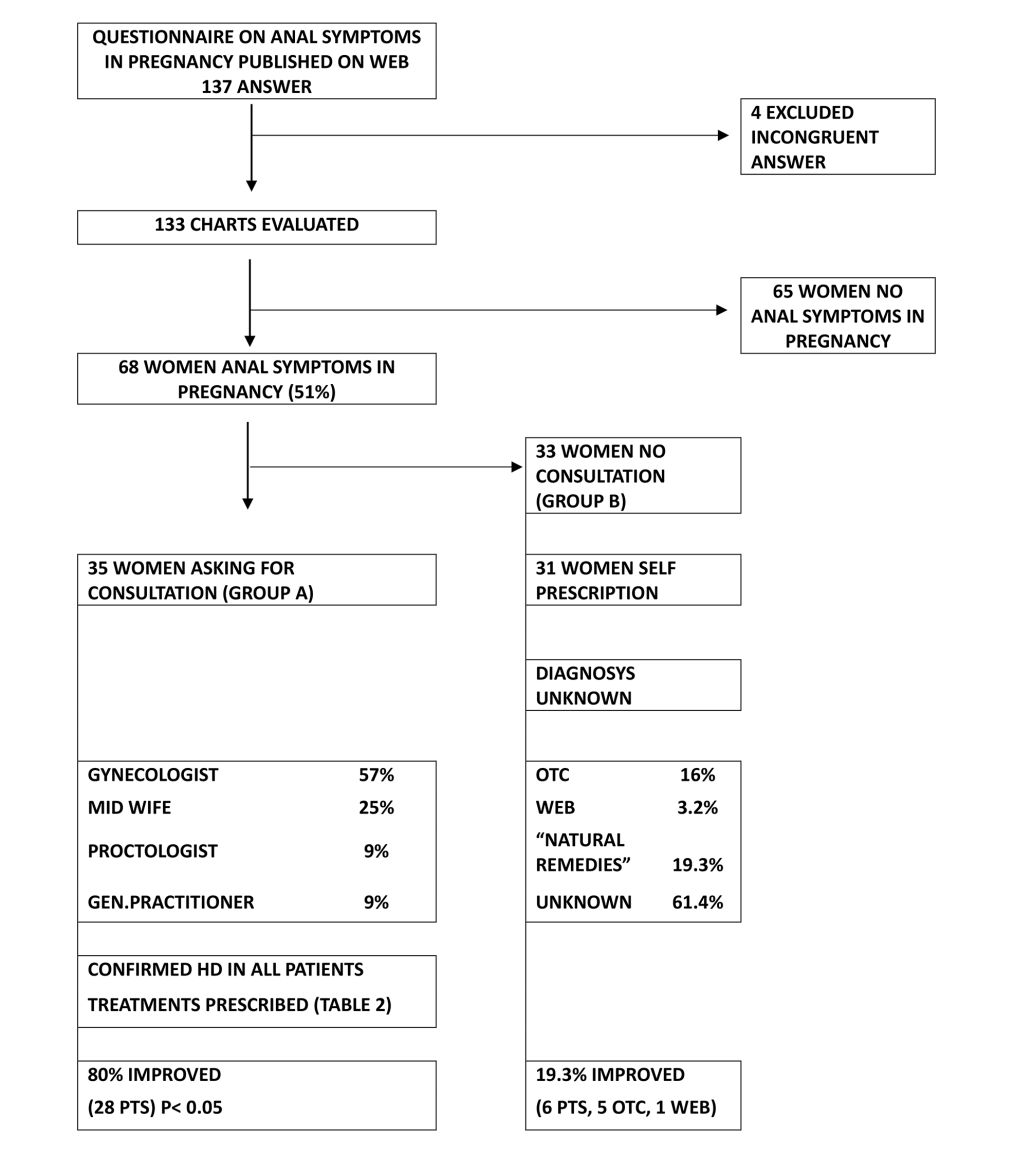
Flow-chart of the study showing some results of answers to questionnaire and treatments adopted. Consultation of specialists and adoption of prescription resulted in a significant improvement of symptoms (80% vs. 19.3%, *p* < 0.05)

Out of 68 women with diagnosis of HD, 35 (Group A, 51.4%) looked for medical consultation, asking for mainly the gynecologist (57%), the midwife (25%), the proctologist or the general practitioner (9% each). The flow-chart in Fig. 1 summarizes the study and part of the results.

The remaining 33 patients (Group B 48.6%) reported self-embarrassment as the reason for not doing a visit in 54.5% of the cases (Table 1). In 30.3% of these, symptoms resolved spontaneously after a short time thus making medical consultation unnecessary.

On the overall, 66 patients affected by symptoms of HD out of 68 adopted for medical treatment and this makes up 97% of the cases. Treatments were adopted either by those women of Group A, seen by a professional [35] and by 31 out of 33 of the Group B who were not examined, indicating a high percentage of self-diagnosis, self-prescription and/or adoption of “popular/natural” remedies or Over the Counter (OTC) products. Table 2 details the type of treatment according to the specialist consulted. However, out of 66 women undergoing treatment, only 51.5% reported a significant improvement, 80% of Group A and only 19.3% of Group B, namely those women relying upon self-prescription (Fig. 1, *p* < 0.05).

**Table 2:** Prescriptions or counselling adopted by different professionals involved in treating HD in pregnancy. Figures are the number of answers recorded at Q8 and Q38

	HEMORRHOID OINTMENT	EPARINOIDS	FLAVONOIDS	LAXATIVES	DIET	PHISYCAL ACTIVITY	SITZ BATHS	NATURAL REMEDIES
GYNECOLOGIST	10	1	1	2	7	-	-	-
MIDWIFE	1	-	1	-	2	4	6	7
PROCTOLOGIST	2	1	-	-	2	2	-	-
GENERAL PRACTITIONER	3	-	1	-	-	2	-	-
PHARMACIST	5	-	9	-	-	-	-	-
INTERNET	-	-	-	-	1	1	1	-

Surgery was not offered to any patient answering to our questionnaire. Before pregnancy normal bowel function or constipation were equally reported by those pregnant women complaining of HD and those who did not. Similarly with the occurrence of pregnancy, the presence of normal bowel function, constipation or diarrhea did not differ significantly between the two groups of pregnant women. Finally, constipation, low fiber and liquid intake, sedentary life, prolonged time on the toilet did not show to be significantly associated to HD in pregnancy with respect to those women not reporting any anal symptom (Table 3). Of the 68 women complaining of HD in pregnancy, a previous diagnosis/treatment of HD was reported by 54.4%; conversely in the group of 65 women not affected by HD in pregnancy, a history of previous HD was reported in 18,4% only (Table 3, OR 5.2, *p* < 0.001). This showed to be the only significant risk factor.

**Table 3:** Risks factors of occurrence of HD in pregnant women complaining of perianal symptoms and in those not complaining

RISK FACTOR N.O (%)	Complaint of HD in pregnancy		OR	CI 95%	*P*
	YES 68 PTS (51)	NO 65 PTS (49)			
PRESENCE OF CONSTIPATION					
YES	32 (47)	24 (36.9)	1.5	1.55-1.44	0.299
NO	26 (53)	41(63.1)			
FIBER INTAKE					
HIGH	62(91.1)	57 (87.6)	0.5	0.27-1.10	0.7
POOR	6 (8.9)	8 (12.3)			
FLUID INTAKE					
> L	52 (76.4)	52 (80)	0.8	0.15-4.2	0.802
< L	16 (23.6)	13 (20)			
PHYSICAL ACTIVITY					
NO	30 (44.1)	34 (52.3)	0.7	0.42-1.15	0.340
YES	38 (55.9)	31(47.6)			
TIME ON THE TOILET					
>5MIN	37 (54.4)	32 (49.2)	1.7	0.53-5.40	0.957
<5MIN	31(45.6)	33(50.8)			
AGE>30 YR	39 (57.3)	25 (38.4)	2.15	0.39-11.80	0.310
AGE<30 YR	29 (42.7)	40 (61.6)			
PREVIOUS HD	37 (54.4)	12 (18.4)			
NO PREVIOUS HD	31 (45.6)	53 (81.5)	5.2	0.03-8.45	0.001

Modality of delivery was predominantly vaginal with very few cesarean or instrumental help, thus not allowing statistical analysis.

## Discussion

HD in pregnancy seems to be promoted by specific etiologic factors [2, 7], different from those occurring in the general population. The clinical picture of HD in pregnancy and immediately after delivery may represent a significant cause of morbidity and poor quality of life. In spite of the standardized approach to such a disease in the general population, as depicted by guidelines of different scientific societies [24–29], measures to relieve symptoms of HD in pregnancy, not rarely very disabling as mentioned above, are based upon a limited pool of empirical suggestions concerning bowel habit, diet, local ointments, fluid intake. The identification of a targeted, etiologic treatment of HD in pregnancy need a full elucidation of epidemiology and pathophysiology of the disease. With this aim we aimed our questionnaire concerning symptoms of HD in pregnancy administered via Google Modules to be answered on a voluntary basis.

The majority of papers in literature are mainly based on interviews or a self-administered questionnaire in hospitalized or care-seeking patients reporting incidence of HD in pregnancy with a wide range from 4 to 10% up to 85% of the cases. This wide range may reflect a selection bias in recruiting patients for the interviews. Self-assessment questionnaires have been widely used for the evaluation of HD in pregnancy with their limitations in data interpretation. In fact, when a questionnaire is administered within hospital or by caregiver, it reaches a selected population i.e. those seeking for medical advice. Social media and dedicated mobile apps concerning maternal health in pregnancy are becoming increasingly popular [21, 22] and they have shown to give great support and improve wellbeing in pregnancy and postpartum [30, 31], contributing to the collection and exchange of data focused on maternal health. The platform of social networks theoretically has the potential to reach a large and really unselected sample of the population. In our work it is the first time to our knowledge that a social network has been used for an interview concerning HD and pregnancy. In our study we observed incidence of HD in pregnancy in about 50% of the patients. This data is placed in between the wide range reported in the literature but is similar to recent research [19]. We can hypothesize that this figure may represents a realistic epidemiologic data due to the unselected nature of our sample. The number of women answering to the questionnaire is not as large as we may have expected by using a social network as in our study. In addition, out of 68 cases we have a confirmed diagnosis of HD performed by a health professional in 35 patients. These are undoubtedly limitations to our investigation, but nevertheless the concordance between the data of the literature concerning the highest peak incidence of HD in pregnancy observed in the 3rd trimester [6] and the results observed in our patient material, (70% in the 3rd trimester and 57% in the 2nd) can reassure about the quality of our data collection. Interestingly, all those well-known etiologic factors triggering HD seem to have little or no effect on pregnant women. Adequate fiber and liquid intake, a normal bowel habit, no strain on the toilet seem not to be protective against the onset HD during pregnancy. In fact, other factors have been found responsible for the occurrence of HD in pregnant women. Increased pelvic pressure, pelvic venous engorgement, vascular effects of progesterone, increased circulating blood volume are believed to be the main determinants in the pathophysiology of HD in pregnancy. Nevertheless, both common sense and scientific literature [32] continue to rely upon the same empirical remedies such as warm sitz baths and increased liquid and fiber intake. A recent multicenter study reaffirms the importance of dietary and behavioral habits in pregnancy and after delivery [33]. In this study, women were advised to eat at regular intervals; increase the intake of fiber and fruit during meals and drink at least 1.5 L of water. The patients also had to exercise for at least 30–60 min, 3–5 times a week. There were specific recommendations for defecation: do not ignore the urge to defecate; spend less than 3 min on the commode, attempting to defecate 30–40 min after eating and in the morning. The data obtained, in this case, showed that a counseling intervention aimed at changing eating and behavioral habits, can significantly reduce the rate of HD in pregnancy. Undoubtedly, such simple recommendations can exert a certain degree of symptom relief but are far from being an etiologically addressed treatment. A positive experience is the one reported by Saleeby [34] who performed hemorrhoidectomy on pregnant women affected by thrombosed hemorrhoids. Twentyfour out of 25 patients reported a great relief of symptoms after removal of thrombosed or gangrenated hemorrhoids. Thus, given the lack of efficacy of empirical conservative methods, it is not surprising that hemorrhoidectomy during pregnancy seems the most effective treatment.

About half of our patients’ complaining of HD (33 out of 68) did not ask any kind of specialist advice, mainly because they felt embarrassed for the visit in first place and, although in less amount, due to the spontaneous remission of the symptoms, thus indicating a self-limiting course of the disease. These two data deserve maximum attention when conducting an in-hospital epidemiological interview/questionnaire; as mentioned above the selection of a patients’ sample among those complaining of severe and persisting symptoms, can exert a bias effect and can be the reason for the over-estimate incidence and severity of the disease as reported in the literature. 97% of the affected patients employed some kind of treatment either following medical prescription or by means of self-medication, in both cases following the mentioned empirical approach. Treatments however, showed efficacy in 34 out of 66 patients (51.5%) who reported a clinical improvement, not surprisingly mainly among those seeking medical advice. This result supports further the need for a targeted etiologic treatment of HD in pregnancy administered in a specialized setting. Midwives tended to rely frequently upon natural remedies of non-proven efficacy such as local packs of herbal mixture or clay, with patients adopting such “natural” treatments reporting no improvement. As for preventive measures, since HD in pregnancy seems to occur in about 50% of cases, a screening proctological visit could be scheduled in the second and third trimester, particularly in those patients with a history of HD before pregnancy since they bear a significantly increased risk of developing HD. Our study shows a lack of information concerning HD and its management in the first month after spontaneous birth, when a worsening of the disease has been reported [35]. This is a clear limitation to the study together with the small sample size. In addition, the diagnosis was based upon symptom and this could include, although in small amount, anal fissure and not only HD. Future investigations focused on epidemiology and management of HD after childbirth and its impact on quality of life may clarify this point. In addition, a parallelism between the development of HD and other perineal problems in post-partum such as stress urinary incontinence or pelvic organ prolapse [36, 37] should be considered and investigated.

## Conclusions

In conclusion, although with the limitation of our study, we may assume that when treating HD in a pregnant woman we are facing a different disease from that occurring in the general population due to the lack of role played by constipation, straining at defecation, diet, etc. When HD occurs in a pregnant woman, a medical consultation with a specialist warrants improvement by means of medical or surgical treatments. A proctologic screening visit may be advisable either in a fertile woman with a history of previous HD planning a pregnancy and in pregnant women at the 2nd and 3rd trimester. Further studies are needed in order to identify the targeted treatment, taking into account however, that in selected cases, surgery can be offered with good results and minimal risks in pregnant women. Recently, the therapy of HD in the general population is finding new therapeutic strategies that in the near future could also be indicated in pregnancy or after delivery [38].

### Electronic supplementary material

Below is the link to the electronic supplementary material.

Supplementary Material 1

## Data Availability

Data are available under reasonable request to the corresponding author.

## Abbreviations

HD: Haemorrhoidal Disease
