# Supplementary material for: Haemorrhoidal disease in pregnancy: results from a self-assessment questionnaire administered by means of a social network
Source: BMC Gastroenterol. 2024 May 2;24:150. doi: 10.1186/s12876-024-03228-5 (PMC11064321; doi:10.1186/s12876-024-03228-5)
Supplement: Supplementary file 1 — Supplementary Material 1 [file 12876_2024_3228_MOESM1_ESM.docx]

**Appendix 1**

**QUESTIONNAIRE:**

1. **HAVE YOU EVER BEEN DIAGNOSED WITH OR SUFFERED FROM ANY ANAL DISEASE BEFORE PREGNANCY? (HEMORRHOIDS, FISSURE, ABSCESS, FISTULA)**

** YES**

** NO**

1. **IF YES, PLEASE INDICATE YOUR SYMPTOM(S)**

** PAIN**

** BLEEDING**

** SWELLING**

** PROLAPSE**

** INCONTINENCE**

** ITCHING**

1. **WHEN DID YOU SUFFER FROM THE SYMPTOMS?**

** AT DEFECATION**

** AFTER DEFECATION**

** ALL DAY LONG**

1. **DID YOU SEEK FOR MEDICAL/MIDWIFERY ADVICE?**

** YES**

** NO**

1. **IF YES, WHICH DIAGNOSIS WAS MADE?**

** HEMORRHOIDS**

** FISSURE**

** THROMBOSED PILES**

** ABSCESS**

1. **IF YOU DECIDED NOT TO SEE A SPECIALIST, WHICH WAS THE REASON?**

** EMBARASSMENT**

** REMISSION**

** LACK OF TIME**

1. **WHICH REMEDIES DID YOU EMPLOY TO TREAT YOUR PROBLEM?**

**________________________________________________________________________________________________________________________________________________________________________________________________________________________________________________**

1. **HOW DID YOU OBTAIN DIAGNOSIS AND TREATMENT?**

** GYNECOLOGIST**

** PROCTOLOGIST**

** MIDWIFE**

** FAMILY DOCTOR**

** PHARMACIST**

** FRIEND**

** INTERNET**

1. **WERE THE REMEDIES SUCCESSFUL?**

** YES**

** NOW**

1. **FOR HOW LONG DID YOU FOLLOW THE TREATMENT?**

** 15 days**

** 30 days**

1. **YOUR AGE AT PREGNANCY**

**>30 YR**

**<30YR**

1. **PHYSICAL ACTIVITY BEFORE PREGNANCY**

** YES**

** NO**

1. **PHYSICAL ACTIVITY DURING PREGNANCY**

** YES**

** NO**

1. **YOUR BOWEL HABIT**

** DAILY**

** TWICE A DAY**

** LESS THAN 3 TIMES A WEEK**

1. **YOUR BOWEL HABIT DURING PREGNANCY**

** DAILY**

** TWICE A DAY**

** LESS THAN 3 TIMES A WEEK**

1. **YOUR DAILY FLUID INTAKE**

**<1LITER**

**> 1 LITER**

1. **WHAT WAS YOUR DAILY FLUID INTAKE DURING PREGNANCY?**

**<1LITER**

**> 1 LITER**

1. **IS YOUR DIET RICH OF VEGETABLE AND FRUITS?**

** YES**

** NO**

1. **DURING PREGNANCY, WAS YOU DIET RICH OF VEGETABLE AND FRUITS?**

** YES**

** NO**

1. **HOW LONG DO YOU STAY ON THE TOILET?**

**Maximum 5 min**

**Longer than 5 min to ensure complete evacuation**

**Longer than 5 min just for reading, relaxing etc…**

1. **HOW LONG DO YOU STAY ON THE TOILET DURING PREGNANCY?**

**Maximum 5 min**

**Longer than 5 min to ensure complete evacuation**

**Longer than 5 min just for reading, relaxing etc**

1. **INDICATE THE SHAPE OF YOUR STOOL ACCORDING TO THE FOLLOWING SCALE**

| **BRISTOL STOOL SCALE** | | **IN PREGN.** | **NOW** |
| --- | --- | --- | --- |
| **TYPE 1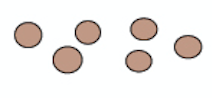** | Separate hard lumps, like nuts- difficult to pass, indicates constipation |  |  |
| **TYPE 2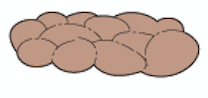** | Sausage-shaped but lumpy, indicates constipation |  |  |
| **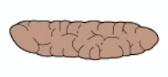TYPE 3** | Sausage-shaped with surface cracks ideal stools as easier to pass |  |  |
| **TYPE 4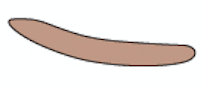** | Sausage-shaped, smooth and soft, ideal stools as easier to pass |  |  |
| **TYPE 5**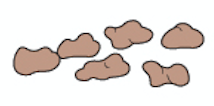 | Soft blobs with clear-cut edges, may indicate diarrhea and urgency |  |  |
| **TYPE 6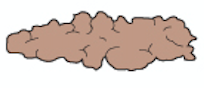** | Fluffy pieces with ragged edges, a mushy stool, may indicate diarrhea and urgency |  |  |
| **TYPE 7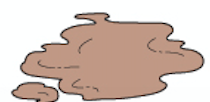** | Watery, no solids, entirely liquid, may indicate diarrhea and urgency |  |  |

1. **PLEASE INDICATE THE TYPE OF DELIVERY**

** SPONTANEOUS VAGINAL**

** OPERATIVE VAGINAL**

** CESAREAN SECTION**

** INDUCED VAGINAL**

1. **DID YOU HAVE EPIDURAL ANALGESIA?**

** YES**

** NO**

** CESAREAN**

1. **DID THE PERSONNEL ASSISTING YOU PRACTICE MANOUVRES AT DELIVERY?**

** YES**

** NO**

** CESAREAN**

1. **IF YES, PLEASE INDICATE WHICH PROCEDURE (S):**

** NONE**

** RUPTURE OF THE SAC**

** OXYTOCINE**

** PUSHING TH ABDOMEN (KRISTELLER)**

** CESAREAN**

1. **DURING PREGNANCY, YOU WERE FOLLOWED BY**

** FAMILY DOCTOR**

** GYNECOLOGIST**

** MIDWIFE**

** HOSPITAL**

1. **DID YOU SUFFER OF HEMORRHOIDS DURING PREGNANCY?**

** YES**

** NO**

1. **IF YES, PLEASE INDICATE THE PERIOD**

** 1^ST^ TRIMESTER**

** 2^ND^ TRIMESTER**

** 3^RD^ TRIMESTER**

** AT DELIVERY**

** BACK HOME**

1. **PLEASE INDICATE THE PREVAILING SYMPTOM(S)**

** PAIN**

** BLEEDING**

** SWELLING**

** PROLAPSE**

** ITCHING**

** SOILING**

1. **IN PRESENCE OF SUCH SYMPTOMS HOW WAS YOUR BOWEL FUNCTION?**

** LESS THAN 3 TIMES A WEEK**

** DAILY**

** DIARRHOEA**

1. **PLEASE INDICATE POSITION DURING LABOUR**

** SUPINE**

** FRE STANDING**

** NONE, ELECTIVE CESAREAN**

1. **PLEASE INDICATE POSITION DURING DELIVERY**

** SUPINE**

** LEFT OR RIGHT SIDE**

** SQUATTING**

** ALL FOURS POSITION**

** BIRTH STOOL**

** WATER**

1. **PLEASE INDICATE THE TIMING OF PUSH**

**<60 MIN**

**> 60 MIN**

**>120MIN**

** CESAREAN**

1. **HOW DID YOU PUSH DURING THE DELIVERY?**

** WHENEVER I FELT I HAD TO**

** WHENEVER I WAS TOLD TO**

** CESAREAN**

1. **WHEN HEMORRHOIDS FIRST APPEARED, WHO MADE THE DIAGNOSIS AND WHO WAS IN CHARGE OF THE TREATMENT?**

** GYNECOLOGIST**

** PROCTOLOGIST**

** MIDWIFE**

** FAMILY DOCTOR**

1. **IF YOU DECIDED NOT TO SEE A SPECIALIST, WHICH WAS THE REASON?**

** EMBARASSMENT**

** REMISSION**

** LACK OF TIME**

1. **WHICH REMEDIES WERE PRESCRIBED FOR TREATING YOUR PROBLEM?**

**________________________________________________________________________________________________________________________________________________________________________________________________________________________________________________**

1. **WERE THEY EFFECTIVE?**

** YES  NOT**
